# Putative progressive and abortive feline leukemia virus infection outcomes in captive jaguarundis (*Puma yagouaroundi*)

**DOI:** 10.1186/s12985-017-0889-z

**Published:** 2017-11-17

**Authors:** Claudia Filoni, A. Katrin Helfer-Hungerbuehler, José Luiz Catão-Dias, Mara Cristina Marques, Luciana Neves Torres, Manfred Reinacher, Regina Hofmann-Lehmann

**Affiliations:** 1 Institute of Biosciences, Department of Microbiology and Immunology, São Paulo State University (UNESP), Campus Botucatu, Rua Professor Doutor Antonio Celso Wagner Zanin SN, Botucatu, São Paulo 18618-689 Brazil; 2 Vetsuisse Faculty, Clinical Laboratory and Center for Clinical Studies, University of Zurich, Winterthurerstrasse 260, 8057 Zurich, Switzerland; 3 School of Veterinary Medicine and Animal Sciences, Department of Pathology, University of São Paulo (USP), Av. Prof. Orlando Marques de Paiva 87, São Paulo, 05508-270 São Paulo Brazil; 4 Fundação Parque Zoológico de São Paulo (FPZSP), Av. Miguel Stéfano 4241, São Paulo, São Paulo 04301-905 Brazil; 5 School of Veterinary Medicine and Animal Sciences, Veterinary Hospital (HOVET), University of São Paulo (USP), Av. Prof. Orlando Marques de Paiva 87, São Paulo, 05508-270 São Paulo Brazil; 6 Institute of Veterinary Pathology, University of Giessen, Frankfurter Strasse 96, Giessen, 35392 Germany; 7 Vetsuisse Faculty, Clinical Laboratory and Center for Clinical Studies, University of Zurich, Winterthurerstrasse 260, 8057 Zurich, Switzerland

**Keywords:** Retrovirus, FeLV-A, enFeLV, Intestinal B-cell lymphoma, qPCR, RT-qPCR, Wild felids

## Abstract

**Background:**

Feline leukemia virus (FeLV) is an exogenous gammaretrovirus of domestic cats *(Felis catus)* and some wild felids. The outcomes of FeLV infection in domestic cats vary according to host susceptibility, virus strain, and infectious challenge dose. Jaguarundis (*Puma yagouaroundi)* are small wild felids from South and Central America. We previously reported on FeLV infections in jaguarundis. We hypothesized here that the outcomes of FeLV infection in *P. yagouaroundi* mimic those observed in domestic cats. The aim of this study was to investigate the population of jaguarundis at Fundação Parque Zoológico de São Paulo for natural FeLV infection and resulting outcomes.

**Methods:**

We investigated the jaguarundis using serological and molecular methods and monitored them for FeLV-related diseases for 5 years. We retrieved relevant biological and clinical information for the entire population of 23 jaguarundis held at zoo. *Post-mortem* findings from necropsies were recorded and histopathological and immunohistopathological analyses were performed. Sequencing and phylogenetic analyses were performed for FeLV-positive samples. For sample prevalence, 95% confidence intervals (CI) were calculated. Fisher’s exact test was used to compare frequencies between infected and uninfected animals. *P*-values <0.05 were considered significant.

**Results:**

In total, we detected evidence of FeLV exposure in four out of 23 animals (17%; 95% CI 5–39%). No endogenous FeLV (enFeLV) sequences were detected. An intestinal B-cell lymphoma in one jaguarundi was not associated with FeLV. Two jaguarundis presented FeLV test results consistent with an abortive FeLV infection with seroconversion, and two other jaguarundis had results consistent with a progressive infection and potentially FeLV-associated clinical disorders and *post-mortem* changes. Phylogenetic analysis of *env* revealed the presence of FeLV-A, a common origin of the virus in both animals (100% identity) and the closest similarity to FeLV-FAIDS and FeLV-3281 (98.4% identity), originally isolated from cats in the USA.

**Conclusions:**

We found evidence of progressive and abortive FeLV infection outcomes in jaguarundis, and domestic cats were probably the source of infection in these jaguarundis.

## Background

Jaguarundis (*Puma yagouaroundi)* are small diurnal felids that have several unpatterned color morphs – brownish-black, gray and reddish-yellow fur – and are protected across most of their range. The species occurs at low densities and has a decreasing population in the wild, despite being widely distributed throughout South and Central America and occupying a broad range of habitats [1]. Mainly due to displacement from nature and, to a lesser extent, to captive breeding, jaguarundis are commonly found in zoos and similar captive settings in Brazil [2].

The feline leukemia virus (FeLV) is an exogenous, oncogenic, immunosuppressive gammaretrovirus that can establish persistent infections in domestic cats *(Felis catus)* [3]. The prevalence of FeLV ranges from 1% to 8% in healthy cats almost everywhere in the world and is usually higher when sick cats are included [4]. FeLV is naturally transmitted by oronasal exposure to virus-containing secretions, mainly saliva, but also in feces and urine [5–7]. The effects of FeLV are cytoproliferative diseases including lymphomas and myeloproliferative disorders; degenerative illnesses, such as anemia and leukopenia; and immunosuppressive diseases associated with opportunistic infections [3, 8].

FeLV infections tend to be rare or absent in many nondomestic felid species [9–14], except for the European wildcat *(Felis silvestris silvestris),* a species very closely related to the domestic cat, in which FeLV appears to be endemic [15–17]. However, documentation of FeLV is becoming more common in wild felid species less closely related to the genus *Felis*, which highlights the omnipresent threat that FeLV represents to the conservation of wild felids worldwide. FeLV has been shown to represent a major threat to the survival of critically endangered populations of Iberian lynxes *(Lynx pardinus)* in Europe [18, 19] and to Florida panthers *(Puma concolor coryi)* in North America [20, 21]. In Brazil, antibodies against FeLV have been detected in two free-ranging pumas *(Puma concolor)* and two jaguarundis; FeLV DNA was detected in the following captive felids: an ocelot *(Leopardus pardalis*), an oncilla *(Leopardus tigrinus)* and two jaguarundis [22–24].

For domestic cats, the detection of the structural viral protein FeLV p27 in serum or plasma is used as a marker of infection and, in most cases, as a parameter for viremia. The outcomes of FeLV infection vary according to infectious challenge dose, route of challenge, and possibly host susceptibility and virus strain. The classification of FeLV outcomes in the domestic cat has been refined using sensitive molecular assays that detect and quantify proviral FeLV DNA and viral FeLV RNA, in addition to traditional serological and virological methods [25–30]. In this regard, the main host response categories of FeLV infection in domestic cats have been redefined as abortive, regressive, and progressive infection. In short, those cats that abort infection do not show any evidence of virus infection, except for seroconversion. Cats with regressive infection overcome viremia after an undetectable or transient initial phase by means of efficient cellular and humoral immune responses. These cats seroconvert, permanently harbor low to moderate FeLV proviral loads integrated in mononuclear cells, i.e., lymphocytes, and may or may not clear their plasma viral RNA loads [25, 27, 31]. Cats with progressive infection are constantly viremic, have low or no antibodies to FeLV, show elevated FeLV proviral and viral loads in peripheral blood cells and plasma and may develop FeLV-associated disease [25, 26, 28, 29].

In previous surveillance work, we detected FeLV infection in two captive-born jaguarundis (#1 and #4) as well as previous exposure to FeLV in two other captive-born jaguarundis (#2 and #22) among a population of 23 jaguarundis held at Fundação Parque Zoológico de São Paulo (FPZSP), Brazil [23]. Jaguarundis #1 and #4 tested positive for FeLV p27 antigen by sandwich ELISA. FeLV proviral DNA was detected in blood from both animals by quantitative real-time polymerase chain reaction (qPCR). The sampling for this previous study occurred between 2003 and 2004 (Table 1).

**Table 1:** Investigation of feline leukemia virus (FeLV) for captive jaguarundis *(Puma yagouaroundi)* and putative infection outcomes

Animal	Zoo registry (CAD)		FeLV serological tests					FeLV molecular tests^a^						enFeLV	Putative infection outcome
			Antibody		Antigen			Viral RNA			Proviral DNA				
		Indirect ELISA p45^b^	Indirect ELISA p15E^c^	Western blotting^b^	Sandwich ELISA^b^	Snap test^b^	IHC^d^ (Tissues)	Whole blood or buffy coat	Saliva	Tissues	Whole blood or buffy coat^b^	Saliva	Tissues	Whole blood or buffy coat	
#1	20761	Neg^e^	Neg	Neg	Pos^f^	Pos	Pos	Pos	Pos	Pos	Pos	Pos	Pos	Neg	Progressive
#2	20762	Pos^g^	Nt^h^	Pos	Neg	Neg	Nt	Neg	Nt	Nt	Neg	Nt	Nt	Neg	Abortive with seroconversion
#3	21566	Neg	Neg	Neg	Neg	Neg	Nt	Neg	Nt	Nt	Neg	Nt	Nt	Neg	Non-infected
#4	21810	Neg	Neg	Neg	Pos	Neg	Pos	Pos	Nt	Nt	Pos	Nt	Nt	Neg	Progressive
#5	22096	Neg	Neg	Neg	Neg	Neg	Neg	Neg	Nt	Neg	Neg	Nt	Neg	Neg	Non-infected
#6	23120	Neg	Nt	Neg	Neg	Neg	Nt	Neg	Nt	Nt	Neg	Nt	Nt	Neg	Non-infected
#7	23851	Neg	Nt	Neg	Neg	Neg	Nt	Neg	Nt	Nt	Neg	Nt	Nt	Neg	Non-infected
#8	23911	Neg	Nt	Neg	Neg	Neg	Nt	Neg	Nt	Nt	Neg	Nt	Nt	Neg	Non-infected
#9	23956	Neg	Nt	Neg	Neg	Neg	Nt	Neg	Nt	Nt	Neg	Nt	Nt	Neg	Non-infected
#10	24142	Neg	Nt	Neg	Neg	Neg	Nt	Neg	Nt	Nt	Neg	Nt	Nt	Neg	Non-infected
#11	24189	Neg	Nt	Neg	Neg	Neg	Nt	Neg	Nt	Nt	Neg	Nt	Nt	Neg	Non-infected
#12	24226	Neg	Nt	Neg	Neg	Neg	Nt	Neg	Nt	Nt	Neg	Nt	Nt	Neg	Non-infected
#13	24227	Neg	Nt	Neg	Neg	Neg	Nt	Neg	Nt	Nt	Neg	Nt	Nt	Neg	Non-infected
#14	24958	Neg	Neg	Neg	Neg	Neg	Neg	Neg	Nt	Nt	Neg	Nt	Nt	Neg	Non-infected
#15	25244	Neg	Nt	Neg	Neg	Neg	Nt	Neg	Nt	Nt	Neg	Nt	Nt	Neg	Non-infected
#16	25845	Neg	Nt	Neg	Neg	Neg	Nt	Neg	Nt	Nt	Neg	Nt	Nt	Neg	Non-infected
#17	25846	Neg	Neg	Neg	Neg	Neg	Nt	Neg	Nt	Nt	Neg	Nt	Nt	Neg	Non-infected
#18	26005	Neg	Nt	Neg	Neg	Neg	Nt	Neg	Nt	Nt	Neg	Nt	Nt	Neg	Non-infected
#19	27088	Neg	Nt	Neg	Neg	Neg	Nt	Neg	Nt	Nt	Neg	Nt	Nt	Neg	Non-infected
#20	27155	Neg	Nt	Neg	Neg	Neg	Nt	Neg	Nt	Nt	Neg	Nt	Nt	Neg	Non-infected
#21	27156	Neg	Nt	Neg	Neg	Neg	Nt	Neg	Nt	Nt	Neg	Nt	Nt	Neg	Non-infected
#22	27689	Neg	Nt	Pos	Neg	Neg	Nt	Neg	Nt	Nt	Neg	Nt	Nt	Neg	Abortive with seroconversion
#23	28018	Neg	Nt	Neg	Neg	Neg	Nt	Neg	Nt	Nt	Neg	Nt	Nt	Neg	Non-infected
Positive results/total tested		1/23	0/6	2/23	2/23	1/23	2/4	2/23	1/1	1/2	2/23	1/1	1/2	0/23	

^a^Viral RNA and proviral DNA detection and quantification by RT-qPCR and qPCR, respectively [30]; enFeLV: investigation of endogenous proviral FeLV by qPCR [44]. ^b^Previously published test results [23]: Indirect ELISA – detection of serum or plasma antibodies against FeLV p45 [64]; Western blotting – detection of antibodies against FeLV proteins gp70, p58, p27, p15, and p12; blots are judged positive if they contain antibodies to at least three of these antigens [65]; sandwich ELISA – FeLV p27 antigen investigated in serum or plasma [66]; Snap test – FeLV p27 antigen measured in serum or fresh blood by Snap™ Combo FeLV Antigen/FIV Antibody Test Kit (IDEXX Laboratories, Inc., Westbrook, Maine 04092, USA); qPCR – proviral DNA detection in whole blood and buffy coat [30]. ^c^Indirect ELISA – detection of plasma antibodies against the FeLV transmembrane protein p15E [32, 33]. Cutoff values were OD = 0.163. ^d^IHC = FeLV antigen immunohistochemical analysis [35]. ^e^Neg Negative test result. ^f^Pos Positive test result. ^g^Pos in indirect ELISA for detection of antibodies against FeLV p45 was 94% of the positive control used. ^h^Nt not tested

By analogy, we hypothesized here that FeLV infection in *P. yagouaroundi* mimics the main outcomes observed for the domestic cat. Thus, the aim of this study was to perform additional serological and molecular tests and monitor the population of jaguarundis at FPZSP for FeLV infection and development of FeLV-related diseases for 5 years (2003–2007).

## Methods

### Animals and sample collections

We retrieved relevant biological and clinical information for the entire population of 23 jaguarundis held at FPZSP, from birth or admission of the animals into the zoo until 2007. Most animals were mature adults (*n* = 16), five were immature and two were geriatric; kittens were absent. Three jaguarundis were wild born, while 20 were captive born at FPZSP. Six females (two of wild origin) and three zoo-born males were reproductively active. Eleven animals from the population were full siblings, and 16 were half siblings. The females (*n* = 9) averaged 4.28 ± 0.57 kg in body weight, and the males (*n* = 14) averaged 5.08 ± 0.49 kg in body weight. Detailed biological data such as age, sex, weight, origin (captive or wild born) and the parental history for the 23 jaguarundis are presented in Table 2.

**Table 2:** Biological data of the studied population of 23 jaguarundis *(Puma yagouaroundi)*

Animal	CAD	Sex	Weight (kg)	Origin	Sampling date	Date of birth/zoo entrance^a^	Age in months	Age group^b^	Father (CAD)	Mother (CAD)
#1	20761	male	5.130	captive born	23/06/2004	24/06/1993	132	geriatric	19914	15245
#2	20762	female	4.505	captive born	26/11/2003	24/06/1993	125	geriatric	19914	15245
#3	21566	female	4.835	wild born	10/12/2003	01/05/1994^a^	115	mature adult	wild born	wild born
#4	21810	male	3.985	captive born	23/06/2004	27/12/1994	114	mature adult	wild born	wild born
#5	22096	male	5.165	captive born	23/06/2004	17/08/1995	106	mature adult	19914	15245
#6	23120	female	5.320	captive born	26/11/2003	09/03/1997	80	mature adult	17406	22097
#7	23851	female	4.250	captive born	03/12/2003	20/06/1998	66	mature adult	17406	20762
#8	23911	male	5.505	captive born	10/12/2003	15/08/1998	64	mature adult	unknown^c^	18544
#9	23956	male	5.260	captive born	10/12/2003	14/09/1998	63	mature adult	17406	unknown^c^
#10	24142	female	4.315	wild born	26/11/2003	01/12/1998^a^	59	mature adult	wild born	wild born
#11	24189	male	5.925	captive born	26/05/2004	05/01/1999	64	mature adult	19914	23120
#12	24226	male	4.925	captive born	25/05/2004	16/02/1999	63	mature adult	19914	21566
#13	24227	male	4.920	captive born	03/12/2003	16/02/1999	58	mature adult	19914	21566
#14	24958	male	5.300	captive born	30/06/2004	02/03/2000	51	mature adult	23119	21566
#15	25244	female	3.685	captive born	26/11/2003	05/11/2000	36	mature adult	23956	24142
#16	25845	male	5.665	captive born	26/05/2004	06/04/2001	37	mature adult	22096	23851
#17	25846	male	4.490	captive born	26/05/2004	06/04/2001	37	mature adult	22096	23851
#18	26005	female	4.105	wild born	19/11/2003	12/06/2001^a^	29	mature adult	wild born	wild born
#19	27088	male	4.765	captive born	03/12/2003	07/01/2002	23	immature adult	23119	25244
#20	27155	male	4.755	captive born	10/12/2003	20/02/2002	22	immature adult	23956	24142
#21	27156	female	4.090	captive born	26/11/2003	20/02/2002	21	immature adult	23956	24142
#22	27689	male	5.325	captive born	03/12/2003	01/09/2002	15	immature adult	22096	23851
#23	28018	female	3.410	captive born	19/11/2003	15/03/2003	8	immature adult	21810	20762

Notes: FeLV-infected (#1, #4) and FeLV seropositive (#2, #22) jaguarundis are identified in bold. ^a^For the wild-born jaguarundis (#3, #10 and #18), the dates refer to entrance in the zoo as their dates of birth are unknown. ^b^Immature adults (*n* = 5) were defined as jaguarundis in the age range of 6 months ≤ age < 24 months for males and 6 months ≤ age < 36 months for females; mature adults (*n* = 16) were defined as those in the age range of 24 months ≤ age < 10 years (120 months) for males and 36 months ≤ age < 10 years for females; geriatric jaguarundis (*n* = 2) were those aged ≥10 years for both sexes. ^c^For two captive-born jaguarundis (#8, #9), the identities of the father and the mother, respectively, were not available

For hematological analysis, several laboratories were used over time, and registries of results from the animals were not completed and standardized. All jaguarundis were vaccinated regularly against feline herpesvirus 1 (FHV-1), feline calicivirus (FCV), feline parvovirus (FPV) and rabies lyssavirus. We monitored the potential development of FeLV-related diseases through clinical data provided by the veterinarian staff at the zoo and registered at the zoo archives.

Serological and molecular FeLV tests were performed, in addition to those previously published [23], using samples that had been previously obtained or additional samples collected throughout the observation period. FeLV tests previously performed included p27 FeLV antigen detection by a sandwich ELISA and the commercial immunoassay Snap™ Combo FeLV Antigen/FIV Antibody Test Kit (IDEXX Laboratories Inc., Westbrook, ME, USA), detection of antibodies against the recombinant non-glycosylated form of the FeLV gp70 surface glycoprotein (p45) by indirect ELISA, detection of antibodies against FeLV p12, p15, p27, p58, and gp70 antigens by Western blot and detection of FeLV proviral DNA by real-time PCR. We investigated the presence of antibodies to FeLV transmembrane protein p15E in serum samples from six jaguarundis (#1, #3, #4, #5, #14 and #17) using the previously described assay [32, 33].

Six jaguarundis (#1, #2, #4, #5, #10 and #14) died between 2005 and 2008 and underwent necropsy at the zoo; for these jaguarundis, relevant clinical and *post-mortem* findings from necropsies were recorded, and several tissues were collected for analyses. Fragments of tissues from these animals were fixed in 10% formalin, embedded in paraffin wax and sectioned. The sections were stained with hematoxylin-eosin (HE) on glass slides for histopathological analyses. Tissue specimens from animals #1 and #5 (bone marrow, mesenteric lymph node and spleen) were also stored at ≤ −70 °C. A saliva specimen from animal #1 was collected at first sampling using a sterile cotton swab immersed in virus transport media, which consisted of phosphate-buffered saline (PBS) balanced salt solution supplemented with 0.5% bovine albumin (BSA), antimicrobial agents (200 U/mL penicillin G, 200 U/mL streptomycin, 25 μg/mL fungizone and 6 μg/mL gentamycin) and was kept at ≤ −70 °C. No additional material could be obtained from animal #2.

### Immunohistology

To clarify the histogenesis of an intra-abdominal mass of tissue that developed in jaguarundi #5, we performed immunohistologic assays by the streptavidin-biotin-peroxidase complex technique with a commercial immunoperoxidase kit (LSAB kit, Dako, Glostrup, Denmark) according to the manufacturer’s protocol. We used silanized microscope slides to produce histologic slides, which were processed by usual deparaffination techniques in xylol and hydrated in alcohols (absolute, 95%, 70%, and distilled water). A microwave antigen retrieval technique using EDTA buffer, pH 9.0, was applied [34]. The sections were incubated overnight at 4 °C with primary antibodies against the T-cell marker CD3 (rabbit polyclonal antibodies A0452; Dako; diluted 1 in 500), the B-cell marker CD79 (mouse monoclonal antibodies; clone HM57; M7051; Dako; diluted 1 in 500), and vimentin (mouse monoclonal antibodies; M0725; Dako; diluted 1 in 100) as well as antibodies against proliferating cell nuclear antigen antibodies (PCNA) (mouse monoclonal antibodies; M0879; Dako; diluted 1 in 800). For all reagents, negative controls were generated by substituting the primary antibody with a class-matched immunoglobulin. These procedures were conducted using the logistics of the Department of Pathology, School of Veterinary Medicine and Animal Sciences, University of São Paulo (USP), São Paulo, SP, Brazil.

We investigated the presence of FeLV antigens in several paraffin-embedded tissues from the deceased jaguarundis #1, #4, #5 and #14. This investigation was carried out using the methods and logistics of the Institute of Veterinary Pathology, University of Giessen, Giessen, Germany [35].

### Extraction of nucleic acids

Total nucleic acid (TNA) was extracted from 100 μL of EDTA-anticoagulated whole blood or buffy coat or from 200 μL of EDTA-anticoagulated plasma using the MagNA Pure LC Total Nucleic Acid Isolation Kit (Roche Diagnostics, Rotkreuz, Switzerland). TNA was eluted into 100 μL of elution buffer and stored at −70 °C until PCR testing was performed. RNA was extracted from the saliva, plasma samples collected from jaguarundi #1 and from serum samples from jaguarundis #1 and #4 using the QIAmp® Viral RNA Mini Kit (Qiagen). gDNA and RNA were extracted from tissues (bone marrow, mesenteric lymph node and spleen) from animals #1 and #5 upon necropsy, as previously described [36, 37]. During all extractions, negative controls consisting of 100 μL of PBS were concurrently prepared with each batch of samples to monitor for cross-contamination.

### Molecular tests for FeLV

FeLV viral RNA loads and proviral loads were investigated using real-time RT-PCR and PCR and primers targeting the U3 region of exogenous FeLV, as previously described [30]. For tissue samples, the total copy numbers detected per reaction were normalized to the glyceraldehyde 3-phosphate dehydrogenase (GAPDH) gene as described [38].

FeLV subgroups A, B and C were investigated in the bone marrow, mesenteric lymph node, and spleen of the jaguarundi #1 and in the serum and plasma of jaguarundi #4 by conventional PCR using the FeLV-A specific primers RB59 and RB17; the FeLV-B specific primers RB53 and RB17 and the FeLV-C specific primers RB58 and RB47 as described [39, 40].

### Feline immunodeficiency virus (FIV) serology and molecular assays

Serum from jaguarundi #5 was tested for the presence of antibodies against feline immunodeficiency virus (FIV) using a commercial immunoassay Snap™ Combo FeLV Antigen/FIV Antibody Test Kit (IDEXX Laboratories) and by Western blotting, as described [41]. FIV real-time and conventional RT-PCR were performed from whole blood from jaguarundi #5, as previously described [42, 43].

### Endogenous FeLV-like sequences (enFeLV) PCR

TNA samples from the whole blood or buffy coat of all 23 jaguarundis were tested for the presence of endogenous FeLV (enFeLV) by the three qPCR assays enFeLV-U3–1, enFeLV-U3–2, and enFeLV-env, as previously described [44].

### Sequencing of FeLV *env* from jaguarundis #1 and #4

FeLV *env* sequences were analyzed for the FeLV-positive jaguarundis #1 and #4 from TNA of previously collected buffy coats. For comparison, a second sequence obtained from bone marrow of animal #1 was analyzed 1.4 years later, at the time of euthanasia. For the analysis of the full-length FeLV *env* sequences, genomic DNA from these samples was amplified using PCR with the primers 5847F (5′ ACATATCGTCCTCCTGACCAC 3′) and 8197R (5′ GAAGGTCGAACCCTGGTCAACT 3′) [43], yielding an approximately 2´370 bp product. To ensure high-fidelity amplification, the PCR was performed using Phusion polymerase and HF buffer (Finnzyme, Ipswich, UK). PCR products were sequenced by Microsynth (Balgach, Switzerland) after purification using the GenElute PCR Clean-Up Kit (Sigma, Fluka GmbH, Buchs, Switzerland). The jaguarundi FeLV-A *env* sequence was submitted to GenBank [KR349469].

### Phylogenetic analysis

Phylogenetic analyses were conducted using MEGA version 6 [45]. The FeLV *env* sequences were aligned using CLUSTAL W [46]. Bootstrap support (1000 replicates) was calculated by the neighbor-joining (NJ) [47] and maximum parsimony (MP) [48] methods, and results >70% were considered significant [49]. The MP tree was obtained using the subtree-pruning-regrafting (SPR) algorithm [48] with search level 1 in which the initial trees were obtained by the random addition of sequences (10 replicates). All positions containing gaps and missing data were eliminated from the dataset (complete deletion option).

### Statistics

For sample prevalence, 95% confidence intervals (CI) were calculated. Fisher’s exact test was used to compare frequencies between infected and uninfected animals. *P*-values <0.05 were considered significant.

## Results

### Clinical data of all 23 jaguarundis

The population of jaguarundis kept at FPZSP from 2003 to 2007 consisted of 23 captive jaguarundis. The animals presented good general condition at sampling. Twenty animals presented discrete to moderate oral disorders, including one or more of the following conditions: gingivitis, presence of tartar, periodontitis, tooth loss and/or fractured teeth. No oral disorders were registered in three immature adults, #19, #21 and #23. At least once during the experiment, 15 animals presented episodes of diarrhea, 11 jaguarundis demonstrated transitory weight loss and three suffered from dermatological disorders, such as alopecia; for all these clinical conditions, the underlying causes of the problems were unknown. Despite regular deworming, intestinal parasites were detected in 18 animals during the study period. In hematological analysis, most animals (*n* = 16) showed some degree of leukopenia (marked: n = 1; mild: *n* = 8) and leukocytosis (marked: *n* = 2; mild: *n* = 5). Jaguarundi #1, which was FeLV positive, and jaguarundi #2, which was FeLV seropositive, showed mild transient leukocytosis. Several (*n* = 12) animals showed mildly increased packed cell volume (PCV) and two showed markedly increased PCV in at least one sampling. Jaguarundis #1 and #2 showed mildly increased PCV, and jaguarundi #22, which was FeLV seropositive, showed a markedly increased PCV. The increased PCV could be due to dehydration as the animals had been fasted before immobilization.

### Deceased animals

Six jaguarundis (#1, #2, #4, #5, #10 and #14) in the investigated population died from 2005 to 2008. Jaguarundi #1 was euthanized 1.4 years after being diagnosed with FeLV infection [23]. This jaguarundi was euthanized because the zoo could not keep it segregated under good ethological conditions, although the animal was in good clinical condition. Jaguarundi #5 developed a large intra-abdominal mass of tissue about 1 year after testing negative for FeLV. After clinical and ultrasonographic examinations, the animal underwent laparotomy for excision of the mass and died 3 days after surgery due to clinical deterioration. Animals #2, #4, #10 and #14 died naturally during the period of study. For the six deceased animals, clinical conditions and main *post-mortem* findings are presented in Table 3.

**Table 3:** Clinical and necroscopic findings of five jaguarundis (*Puma yagouaroundi)* that died between 2005 and 2008

Animal identification	Origin	Sex	Age category at death	Clinical condition before death	Main *post-mortem* findings
#1	Captive born	Male	Geriatric	Clinically healthy^a^	Hepatitis, pulmonary edema and emphysema, splenic lymphoid depletion, renal tubular degeneration, renal medullary fibrosis, glomerulonephritis, obesity, muscular atrophy of hind limbs
#2	Captive born	Female	Geriatric	Weight loss	Severe membranous proliferative glomerulonephritis, parasitic hemorrhagic enteritis, hepatic steatosis, cardiomyopathy
#4	Captive born	Male	Mature	Weight loss, vomiting, anorexia, diarrhea	Hepatitis, pulmonary infarction, splenic hypoplasia, decreased number and size of spleen germinal centers, membranous proliferative glomerulonephritis, enteritis, adrenal necrosis
#5	Captive born	Male	Mature	Malignant lymphoma, weight loss and clinical deterioration	Hepatitis, pulmonary emphysema, splenic lymphoid depletion and follicular hypoplasia, membranous proliferative glomerulonephritis, renal tubular degeneration, severe enteritis, malignant neoplasia of round cells (intestinal B-cell lymphoma)
#10	Wild	Female	Mature	Weight loss, circulatory shock, convulsion	Severe interstitial histiocytic bronchopneumonia associated with the presence of intralesional protozoa; intense multifocal and coalescent pancreatic fibrosis
#14	Captive born	Male	Mature	Weight loss, diarrhea	Colitis, enteritis, gastritis, lymphopathy, inguinal herniation, ulcers on the tongue

^a^Jaguarundi #1 was recognized to be FeLV positive and shedding FeLV in its saliva; thus, the animal was euthanized while clinically healthy to prevent further spreading of FeLV infection

### Indirect ELISA for p15E

Antibodies against p15E antigen were not detected to a level that is considered positive for privately owned domestic cats and the results from all the jaguarundis tested (#1, #3, #4, #5, #14 and #17) were negative (Table 1).

### FeLV-negative B-cell lymphoma in jaguarundi #5

The excised mass of tissue from jaguarundi #5 weighed approximately 0.25 kg and affected all layers of the wall of the jejunum. In addition, several small foci were present across the mesentery. There was multifocal necrosis as well as intense reactional fibrosis and proliferation of round cells arranged in dense blocks limited by delicate conjunctive septa. The cells were neoplastic and malignant and presented anisokaryosis, anisocytosis, and a high mitotic index. The tumor was found to be an intestinal B-cell lymphoma, as the cells stained positive for a B-cell marker (CD79) but not a T-cell marker (CD3) by immunohistology. No FeLV RNA or provirus was detected in the tissues collected upon necropsy, including tumoral tissues, bone marrow, mesenteric lymph nodes and spleen, and no FeLV antigens were detected in paraffin-embedded tissues by immunohistological analysis (Table 1). Because B-cell lymphomas can also be associated with FIV infection, jaguarundi #5 was tested for FIV by serology and PCR. The animal tested negative for antibodies to FIV in the commercial assay. In the FIV Western blot, one band against p24 was detectable.

### Molecular detection of FeLV-A in two jaguarundis (#1 and #4)

FeLV RNA and proviral DNA were detected in two out of the 23 animals, namely, jaguarundis #1 and # 4. From jaguarundi #1, a saliva sample was available; it was FeLV RNA positive, indicating shedding of FeLV at the time of sampling. FeLV was also demonstrated in the plasma sample collected at a second sampling from jaguarundi #4 upon necropsy 1.4 years later, indicating persistent antigenemia in this animal. For jaguarundis #1 and # 4, respectively, the absolute copy numbers of proviral DNA were 4.26 × 10^7^ and 3.49 × 10^7^ copies of DNA/mL of buffy coat; the absolute copy numbers of viral RNA were 2.63 × 10^8^ and 2.97 × 10^9^ copies of RNA/mL of serum. The results from all FeLV tests are presented in Tables 1 and 4. Furthermore, FeLV could be demonstrated in the tissue samples collected at necropsy from jaguarundi #1, and FeLV antigens were confirmed by immunohistological analysis of paraffin-embedded tissue samples from deceased jaguarundis #1 and #4. For both jaguarundis, #1 and #4, FeLV-A was the only FeLV subtype identified; no FeLV-B or FeLV-C was detected.

**Table 4:** Feline leukemia virus (FeLV) viral and proviral loads from jaguarundis *(Puma yagouaroundi)* presenting putative progressive infection

Animal	Viral load (RNA) by RT-qPCR					Proviral load (DNA) by qPCR				
	Serum^a^	Saliva^b^	Mesenteric lymph node^c^	Bone marrow^c^	Spleen^c^	Buffy coat^a^	Saliva^b^	Mesenteric lymph node^c^	Bone marrow^c^	Spleen^c^
#1	2.63 × 10^8^	1.65 × 10^5^	1.31 × 10^0^	4.74 × 10^0^	1.74 × 10^0^	4.26 × 10^7^	1.88 × 10^3^	2.64 × 10^−1^	5.61 × 10^−1^	3.68 × 10^−1^
#4	2.97 × 10^9^	Nt^d^	Nt	Nt	Nt	3.49 x 10^7^	Nt	Nt	Nt	Nt

^a^Total copy numbers of FeLV RNA or provirus detected per mL of serum or buffy coat, respectively. ^b^Total copy numbers detected per reaction (5 μL of total nucleic acid – TNA); no absolute quantification was possible since the input saliva volume from the swab sample was not known. ^c^Total copy numbers detected per reaction in tissue samples, normalized to the copy number of the glyceraldehyde 3-phosphate dehydrogenase (GAPDH) gene. ^d^Nt not tested; samples were not available

### No detection of enFeLV

No enFeLV was detected in the blood or buffy coat samples from any of the 23 jaguarundis.

### Sequencing and phylogenetic analysis of the jaguarundi FeLV-A

**Fig. 1:**
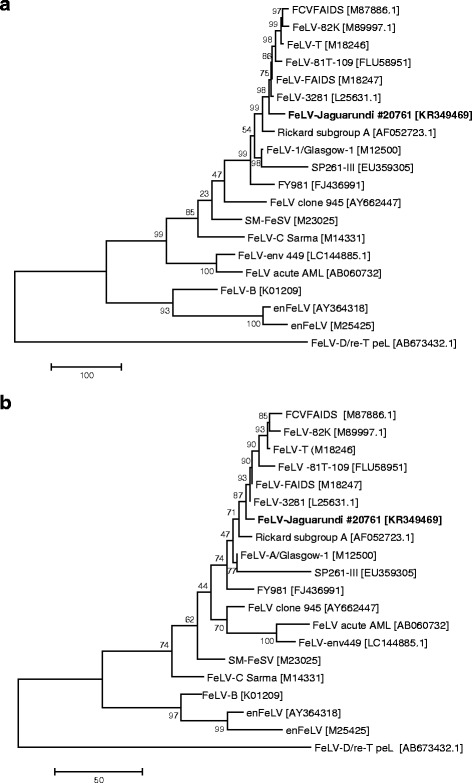
Analysis of the evolutionary relationship using the maximum parsimony (MP) method with 20 sequences. **a** Relationships at the DNA level. Tree #1 out of the 2 most parsimonious trees (length = 1616) is shown. The consistency index is (0.622642), the retention index is (0.696970), and the composite index is 0.515827 (0.433962) for all sites and parsimony-informative sites (in parentheses). There were a total of 1837 positions in the final dataset. **b** Relationships at the protein level. The most parsimonious tree, with length = 637, is shown. The consistency index is (0.750000), the retention index is (0.747664), and the composite index is 0.620901 (0.560748) for all sites and parsimony-informative sites (in parentheses). There were a total of 605 positions in the final dataset. The percentages of replicate trees in which the associated taxa clustered together in the bootstrap test (1000 replicates) are shown next to the branches. The tree is drawn to scale, with the length being relative to the number of changes over the entire sequence. The MP tree was obtained using the subtree-pruning-regrafting (SPR) algorithm with search level 1 in which the initial trees were obtained by the random addition of sequences (10 replicates). All positions containing gaps and missing data were eliminated. Analyses were conducted in MEGA 6 [44–47, 63]

Sequencing of the FeLV isolate retrieved from jaguarundis #1 and #4 revealed one conserved *env* sequence with 100% identity. Similarly, no sequence variation could be detected between the virus present in buffy coat from jaguarundi #1 and the virus detected in bone marrow at the time of euthanasia 1.4 years later in the same animal (100% identity). Phylogenetic analysis conducted by the MP and NJ methods revealed clustering of the jaguarundi *env* with several North American virus strains showing the closest similarity to FeLV-FAIDS [M18247] and FeLV-3281 [L25631], with 98.4% identity (Fig. 1). When the *env* surface unit from the FeLV detected in jaguarundis was compared with those found in Iberian lynxes [EU293175 to EU293194], a sequence identity of approximately 97% was obtained (data not shown).

## Discussion

We detected evidence of FeLV exposure in four (#1, #2, #4 and #22) out of 23 jaguarundis in the FPZSP (17%; 95% CI 5–39%). The remaining 19 jaguarundis were negative for FeLV in all serological and molecular tests performed. The population of jaguarundis presented clinical disorders that were common at FPZSP, as in any other captive setting in Brazil [2, 12], and could be associated with a multitude of causes. Although the FeLV-positive jaguarundis also presented some conditions that could be related to FeLV [8], our data were insufficient to prove a causal association.

Two captive-born male jaguarundis, the geriatric #1 and the mature adult #4, presented serological and molecular FeLV test results similar to the progressive FeLV infection outcome in domestic cats [25]. Jaguarundis #1 and #4 were both antigenemic. Antigenemia is usually used as a measure for viremia in domestic cats and is consistently found in domestic cats with a progressive FeLV infection outcome [26, 28]. Additionally, blood, buffy coat, serum and tissue samples from both jaguarundis were positive for FeLV proviral DNA and viral RNA with high proviral and viral loads, respectively (Table 4). While the presence of proviral DNA attests to the integration of provirus into blood cells, the detection of viral RNA usually indicates replicating virus. Once the infection outcome has been established, high proviral and FeLV RNA loads are characteristically found in cats with progressive infection, while low loads are detected in cats with regressive infection that are provirus positive but not viremic at the time [25, 29, 50]. FeLV proviral and viral RNA loads were measured in the two FeLV-positive jaguarundis #1 and #4. Comparison of the FeLV tissue loads of jaguarundi #1 revealed loads similar to those reported earlier for domestic cats with progressive infection using identical methods. Viral RNA loads in serum from the two FeLV-positive jaguarundis were comparable to those described earlier in persistently antigenemic cats [30]. The viral RNA load in the clinically healthy jaguarundi #1 was slightly lower than that in jaguarundi #4, which showed weight loss, vomiting, anorexia and diarrhea before death. This is also similar to previous findings from a study in cats, comparing viremic healthy and viremic ill cats [51]. Moreover, consistent with findings in domestic cats with a progressive FeLV infection, no antibodies to FeLV antigens were detected in jaguarundis #1 and #4.

FeLV RNA and FeLV provirus were also detected in the saliva of jaguarundi #1. This finding is indicative of virus shedding; thus, jaguarundi #1 was a potential source of FeLV infection to other felids. Jaguarundi #1 was euthanized in good clinical condition as a biosafety measure. However, upon necropsy, histopathologic evaluation revealed splenic lymphoid depletion and muscular atrophy of the hind limbs; these findings may be associated with immunosuppression and neurological impairment in this animal. Jaguarundi #4 died presenting weight loss and gastrointestinal disorders including vomiting, anorexia and diarrhea. Histopathology revealed splenic alterations and evidence of enteritis (Table 3). Although these conditions are commonly associated with FeLV in domestic cats, it was not possible to ascertain whether they were caused by FeLV infection in this case.

Two captive-born jaguarundis, #2 and #22, presented test results similar to those reported for domestic cats with abortive FeLV infection and seroconversion as the only marker of FeLV exposure [28]. The female jaguarundi #2 had a history of direct contact with the two jaguarundis with progressive FeLV infection, #1 and #4: she was a sibling of jaguarundi #1 and had mated with the male #4. This would explain how she was exposed to FeLV, either at the same time as her sibling #1 or during mating with #4. Subsequently, jaguarundi #2 gave birth to jaguarundi #23, who did not show any signs of FeLV exposure (Tables 1, 2, 4). This may well be the case, since the mother, #2, had developed an antibody response and probably had never shed FeLV (abortive infection). It is unknown for how long and at what virus challenge conditions jaguarundi #2 was exposed, as animals were naturally and not experimentally infected in the present study. Overall, we speculate that jaguarundi #2 developed an abortive FeLV infection for similar (and possibly several) reasons that may drive FeLV abortive infections in some domestic cats. The parents of male jaguarundi #22 were the FeLV-negative male jaguarundi #5 and the female #7. Thus, it is unknown at what time point jaguarundi #22 was exposed to FeLV.

Overall, we provide evidence of progressive and abortive FeLV infection in *P. yagouaroundi*. Similar results have been found in Florida panthers *(Puma concolor coryi*), which belong to the felid lineage 6 (genus *Puma*), the same phylogenetic lineage as the jaguarundis [52]. In Florida panthers, infection outcomes resembled those of domestic cats with progressive, regressive and abortive infection (previously persistent, regressive, and latent infection) [20, 21]. We speculate that FeLV infection in jaguarundis is similarly unpredictable, and it is influenced for the same diverse aspects cited above for domestic cats.

Complimentary to the fact that FeLV causes various tumors in domestic cats [53], the literature has reported a FeLV-associated multicentric T-cell lymphoma in a captive non-domestic cheetah *(Acinonyx jubatus)* [54] and non-FeLV-associated T- and B-cell lymphomas in geriatric African lions *(Panthera leo)* [55]. These data motivated the search for possible FeLV involvement in the appearance of the neoplastic intra-abdominal mass in jaguarundi #5. Although jaguarundi #5 had not shown any evidence of FeLV infection *intra vitam* (Table 1), we further investigated whether FeLV antigens or proviruses were present in intestinal lymphoid cells and whether these cells produced FeLV viral RNA locally without it entering the bloodstream – as would be expected in a case of sequestered FeLV infection [4]. However, no FeLV antigens were detected by immunohistochemistry and no FeLV provirus or viral RNA were detected by molecular assays. Infection with FIV, which is a lentivirus associated with lymphomagenesis in domestic cats [56], was not detected by the commercial immunoassay, and only antibodies specific to FIV p24 capsid proteins were detected by Western blot, which may be indicative of an early or very late FIV infection or may have resulted from unspecific cross-reactivity. The FIV RT-PCR assays performed from whole blood were negative; however, this may be due to low viral loads or lack of specificity of the assays due to sequence diversity of different FIV isolates. In conclusion, jaguarundi #5 had developed a non FeLV-associated intestinal B-cell lymphoma; involvement of FIV in the development of the neoplasia cannot completely be ruled out.

Notably, a spleen sample from jaguarundi #5 had been previously analyzed for the presence of the FeLV receptor fTHTR1, which permits the virus to enter the cell. The fTHTR1 complementary DNA (cDNA) from that animal had shown 99% nucleotide and amino acid identity to the fTHTR1 cDNA sequences of the domestic cat and other wild felid species, such as the lynx *(Lynx pardinus)*, African lion *(P. leo bleyenberghi)*, Asiatic lion *(P. leo persica)*, and European wildcat *(F. silvestris silvestris)* [57]. In addition, fTHTR1 was quantified by real-time PCR in a few tissues from two jaguarundis where tissue was available (the viremic jaguarundi #1 and jaguarundi #5, which developed a tumor, but was not FeLV-infected).These limited results showed no significant difference between the fTHTR1 tissue loads in cat and jaguarundi tissue (results not shown). However, it also needs to be mentioned that the real-time PCR assay was designed for the domestic cat THTR1 and not the jaguarundi THTR1. Two-point mutations can be found in the region of the assay: one-point mutation in the middle of the forward primer and one in the probe. Thus, although similar fTHTR1 expression levels were found in jaguarundi tissue compared with cat tissue, we cannot exclude differences in the efficiency of the real-time PCR assay. These findings support the perspective that the first phase of the FeLV virus cycle – viral entry – might be similar among domestic cats and other wild felid species.

We did not detect enFeLV in the jaguarundis by qPCR. This is consistent with earlier data [58] in which enFeLV was not detected in jaguarundis. Accordingly, FeLV-B, which has greater pathogenicity than FeLV-A in domestic cats and arises by recombination of exogenous FeLV-A with enFeLV sequences [59], was not found in the two FeLV-positive jaguarundis #1 and #4. Moreover, the highly virulent FeLV-C was not detected. In FeLV-infected Iberian lynxes, enFeLV sequences were also not detected, but their FeLV-A variants were shown to be highly virulent, suggesting that the mechanisms inducing disease in these wild felids might be distinct from those in domestic cats [19].

The *env* sequence obtained from the FeLV infections of jaguarundis #1 and #4 [KR349469] clustered with the highest identity with the FeLV-FAIDS and FeLV-3281 strains, which represent members of a highly conserved group of horizontally transmitted, minimally pathogenic FeLV-A present in all naturally occurring infections in domestic cats. Both FeLV strains were originally isolated from cats in the United States of America (USA) [60, 61]. Interestingly, the surface unit of the *env* sequence of FeLV detected in the jaguarundis also showed 97% identity to those of the *env* gene of FeLV isolated from the critically endangered Iberian lynxes [EU293175 to EU293194] in Spain, whose small wild population suffered from an outbreak of FeLV with several deaths [18].

Both PCR-positive jaguarundis, #1 and #4, were infected with a FeLV with an identical *env* sequence, indicating a common origin of the virus. It is unknown at what time point each of the jaguarundis became infected. The reproductive success achieved for jaguarundis in captivity is probably a result of the breeding program conceived for small felids at FPZSP [62] and a number of measures for improving the well-being of the animals, including the location of the jaguarundi enclosures in an isolated and forested area of the zoo away from the public. Nonetheless, domestic cats commonly are seen invading the zoo, especially the more isolated areas, and they were probably the source of infection of FeLV for the jaguarundis. Domestic cats are the main reservoir for FeLV worldwide and greatly outnumber FeLV-infected wild felids. Given this, it is prudent to prevent the presence of domestic cats in nature reserves, zoos and other settings with captive wild felids and to reinforce the importance of control measures for FeLV in domestic cats, such as testing and vaccination.

A severe FeLV outbreak occurred in a previously naïve population of Florida panthers *(Puma concolor coryi)* in North America from 2002 to 2005 in which five FeLV antigen-positive panthers died [20, 21]. During a six-month period in 2007, six provirus-positive antigenemic Iberian lynxes *(Lynx pardinus)* died in Spain [18, 19]. In both situations, FeLV vaccination programs were initiated. We recommend safe recombinant subunits or inactivated FeLV vaccines for captive jaguarundis, especially considering that invading domestic cats, potential sources of infection, are a frequent problem faced by zoos. In addition, we recommend the inclusion of FeLV testing in breeding programs for jaguarundis.

## Conclusions

Our findings support our hypothesis that FeLV infection in *P. yagouaroundi* mimics the outcomes observed for the domestic cat. In addition, we found phylogenetic evidence that domestic cats may have been the source of the FeLV-A infection of the jaguarundis. Despite the minimal pathogenicity of FeLV-A for domestic cats, this virus may represent a threat to the jaguarundi population, and regular FeLV testing and vaccination are encouraged.

## References

[1] Caso A, Oliveira T, Carvajal SV (2015). Herpailurus yagouaroundi. The IUCN red list of threatened species.

[2] Adania CH, Silva JCR, Felippe PAN, Cubas ZS, Silva JCR, Catão-Dias JL (2014). Carnivora – Felidae (Onça, Suçuarana, Jaguatirica e Gato-do-Mato). Tratado de animais selvagens: medicina veterinária.

[3] Hoover EA, Mullins JI (1991). Feline leukemia virus infection and diseases. J Am Vet Med Assoc.

[4] Hartmann K (2012). Clinical aspects of feline retroviruses: a review. Viruses.

[5] Francis DP, Essex M, Hardy WD (1977). Excretion of feline leukaemia virus by naturally infected pet cats. Nature.

[6] Gomes-Keller MA, Tandon R, Gönczi E, Meli ML, Hofmann-Lehmann R, Lutz H (2006). Shedding of feline leukemia virus RNA in saliva is a consistent feature in viremic cats. Vet Microbiol.

[7] Gomes-Keller MA, Gönczi E, Grenacher B, Tandon R, Hofman-Lehmann R, Lutz H (2009). Fecal shedding of infectious feline leukemia virus and its nucleic acids: a transmission potential. Vet Microbiol.

[8] Reinacher M (1989). Diseases associated with spontaneous feline leukemia virus (FeLV) infection in cats. Vet Immunol Immunopathol.

[9] Driciru M, Siefert L, Prager KC, Dubovi E, Sande R, Princee F, Friday T, Munson L (2006). A serosurvey of viral infections in lions *(Panthera leo)*, from queen Elizabeth National Park, Uganda. J Wildl Dis.

[10] Thalwitzer S, Wachter B, Robert N, Wibbelt G, Müller T, Lonzer J, Meli ML, Bay G, Hofer H, Lutz H (2010). Seroprevalences to viral pathogens in free-ranging and captive cheetahs *(Acinonyx jubatus)* on Namibian Farmland. Clin Vaccine Immunol.

[11] Ostrowski S, Van Vuuren M, Lenain DM, Durand A (2003). A serologic survey of wild felids from central west Saudi Arabia. J Wildl Dis.

[12] Filoni C, Adania CH, Durigon EL, Catão-Dias JL. Serosurvey for feline leukemia virus and lentiviruses in captive small neotropic felids in São Paulo state, Brazil. J Zoo Wildl Med. 2003;34:65–8. 10.1638/1042-7260(2003)34[0065:SFFLVA]2.0.CO;2 12723802

[13] Hofmann-Lehmann R, Fehr D, Grob M, Elgizoli M, Packer C, Martenson JS, O’Brien SJ, Lutz H (1996). Prevalence of antibodies to feline parvovirus, calicivirus, herpesvirus, coronavirus, and immunodeficiency virus and of feline leukemia virus antigen and the interrelationship of these viral infections in free-ranging lions in East Africa. Clin Diag Lab Immunol.

[14] Blanco K, Peña R, Hernández C, Jiménez M, Araya LN, Romero JJ, Dolz G. Serological detection of viral infections in captive wild cats from Costa Rica. Vet Med. 2011; 10.4061/2011/879029. Internat 879029 10.4061/2011/879029 PMC3087557 21547230

[15] Leutenegger CM, Mislin CN, Sigrist B, Ehrengruber MU, Hofmann-Lehmann R, Lutz H (1999). Viral infections in free-living populations of the European wildcat. J Wildl Dis.

[16] Daniels MJ, Golder MC, Jarrett O, MacDonald DW (1999). Feline viruses in wildcats from Scotland. J Wildl Dis.

[17] Fromont E, Sager A, Léger F, Bourguemestre F, Jouquelet E, Stahl P, Pontier D, Artois M (2000). Prevalence and pathogenicity of retroviruses in wildcats in France. Vet Rec.

[18] Meli ML, Cattori V, Martínez F, López G, Vargas A, Palomares F, López-Bao JV, Hofmann-Lehmann R, Lutz H. Feline leukemia virus and other pathogens as important threats to the survival of the critically endangered Iberian lynx *(Lynx pardinus)*. PLoS One. 2009;4(3):e4744. 10.1371/journal.pone.0004744. 10.1371/journal.pone.0004744 PMC2649436 19270739

[19] Marina ML, Cattori V, Martínez F, López G, Vargas A, Palomares F, López-Bao JV, Hofmann-Lehmann R, Lutz H (2010). Feline leukemia virus infection: a threat for the survival of the critically endangered Iberian lynx *(Lynx pardinus)*. Vet Immunol Immunopathol.

[20] Cunningham MW, Brown MA, Shindle DB, Terrell SP, Hayes KA, Ferree BC, McBride RT, Blankenship EL, Jansen D, Citino SB, Roelke ME, Kiltie RA, Troyer JL, O’Brien SJ. Epizootiology and management of feline leukemia virus in the Florida puma. J Wildl Dis. 2008;44:537–52. 10.7589/0090-3558-44.3.537 PMC3167064 18689639

[21] Brown MA, Cunningham MW, Roca AL, Troyer JL, Johnson WE, O’Brien SJ (2008). Genetic characterization of feline leukemia virus from Florida panthers. Emerg Infect Dis.

[22] Guimaraes AMS, Brandão PE, de Moraes W, Cubas ZS, Santos LC, Villarreal LYB, Robes RR, Coelho FM, Resende M, Santos RCF, Oliveira RC, Yamaguti M, Marques LM, Neto RL, Buzinhani M, Marques R, Messick JB, Biondo AW, Timenetsky J (2009). Survey of feline leukemia virus and feline coronaviruses in captive neotropical wild felids from southern Brazil. J Zoo Wildl Med.

[23] Filoni C, Catão-Dias JL, Cattori V, Willi B, Meli ML, Corrêa SHR, Marques MC, Adania CH, Silva JCR, Marvulo MFV, Neto JSF, Durigon EL, Carvalho VM, Coutinho SD, Lutz H, Hofmann-Lehmann R (2012). Surveillance using serological and molecular methods for the detection of infectious agents in captive Brazilian neotropic and exotic felids. J Vet Diagn Investig.

[24] Filoni C, Catão-Dias JL, Bay G, Durigon EL, Jorge RS, Lutz H, Hofmann-Lehmann R (2006). First evidence of feline herpesvirus, calicivirus, parvovirus, and Ehrlichia exposure in Brazilian free-ranging felids. J Wildl Dis.

[25] Hofmann-Lehmann R, Cattori V, Tandon R, Boretti FS, Meli ML, Riond B, Lutz H. How molecular methods change our views of FeLV infection and vaccination. Vet Immunol Immunopathol. 2008;123:119–123. 10.1016/j.vetimm.2008.01.017 18295346

[26] Hofmann-Lehmann R, Cattori V, Tandon R, Boretti FS, Meli ML, Riond B, Pepin AC, Willi B, Ossent P, Lutz H (2007). Vaccination against the feline leukaemia virus: outcome and response categories and long-term follow-up. Vaccine.

[27] Major A, Cattori V, Boenzli E, Riond B, Ossent P, Meli ML, Hofmann-Lehmann R, Lutz H (2010). Exposure of cats to low doses of FeLV: seroconversion as the sole parameter of infection. Vet Res.

[28] Torres AN, Mathiason CK, Hoover EA (2005). Re-examination of feline leukemia virus: host relationships using real-time PCR. Virology.

[29] Cattori V, Pepin AC, Tandon R, Riond B, Meli ML, Willi B, Lutz H, Hofmann-Lehmann R (2008). Real-time PCR investigation of feline leukemia virus proviral and viral RNA loads in leukocyte subsets. Vet Immunol Immunopathol.

[30] Tandon R, Cattori V, Gomes-Keller MA, Meli ML, Golder MC, Lutz H, Hofmann-Lehmann R (2005). Quantitation of feline leukaemia virus viral and proviral loads by TaqMan real-time polymerase chain reaction. J Virol Methods.

[31] Pepin AC, Tandon R, Cattori V, Niederer E, Riond B, Willi B, Lutz H, Hofmann-Lehmann R (2007). Cellular segregation of feline leukemia provirus and viral RNA in leukocyte subsets of long-term experimentally infected cats. Virus Res.

[32] Boenzli E, Hadorn M, Hartnack S, Huder J, Hofmann-Lehmann R, Lutz H. Detection of antibodies to the feline leukemia virus (FeLV) transmembrane protein p15E: an alternative approach for serological FeLV detection based on antibodies to p15E. J Clin Microbiol. 2014;52:2046–52. 10.1128/JCM.02584-13 PMC4042764 24696026

[33] Nesina S, Helfer-Hungerbuehler AK, Riond B, Boretti FS, Willi B, Meli ML, Grest P, Hofmann-Lehmann R. Retroviral DNA - the Silent Winner: Blood Transfusion Containing Latent Feline Leukemia Provirus Causes Infection and Disease in Naïve recipient cats. Retrovirology 12. BioMed Central. 2015;105 10.1186/s12977-015-0231-z. 10.1186/s12977-015-0231-z PMC4687292 26689419

[34] Hsu SM, Raine L, Fanger H (1981). Use of avidin-biotin-peroxidase complex (ABC) in immunoperoxidase techniques: a comparison between ABC and unlabeled antibody (PAP) procedures. J Histochem Cytochem.

[35] Kipar A, Kremendahl J, Grant CK, von Bothmer I, Reinacher M (2000). Expression of viral proteins in feline leukemia virus-associated enteritis. Vet Pathol.

[36] Helfer-Hungerbuehler AK, Widmer S, Hofmann-Lehmann R. GAPDH pseudogenes and the quantification of feline genomic DNA equivalents. Mol Biol Internat. 2013;587680 10.1155/2013/587680. 10.1155/2013/587680 PMC3655645 23738070

[37] Kessler Y, Helfer-Hungerbuehler AK, Cattori V, Meli ML, Zellweger B, Ossent P, Riond B, Reusch CE, Lutz H, Hofmann-Lehmann R (2009). Quantitative TaqMan real-time PCR assays for gene expression normalisation in feline tissues. BMC Mol Biol.

[38] Leutenegger CM, Mislin CN, Sigrist B, Ehrengruber MU, Hofmann-Lehmann R, Lutz H (1999). Quantitative real-time PCR for the measurement of feline cytokine mRNA. Vet Immunol Immunopathol.

[39] Sheets RL, Pandey R, Jen WC, Roy-Burman P (1993). Recombinant feline leukemia virus genes detected in naturally occurring feline lymphosarcomas. J Virol.

[40] Mathes LE, Pandey R, Chakrabarti R, Hofman FM, Hayes KA, Stromberg P, Roy-Burman P (1994). Pathogenicity of a subgroup C feline leukemia virus (FeLV) is augmented when administered in association with certain FeLV recombinants. Virology.

[41] Lutz H, Arnold P, Hübscher U, Egberink H, Pedersen N, Horzinek MC (1988). Specificity assessment of feline T-lymphotropic lentivirus serology. Zentralbl Veterinarmed B.

[42] Allenspach K, Amacker M, Leutenegger CM, Hottiger M, Hofmann-Lehmann R, Hübscher U, Pistello M, Lutz H (1996). Quantification of proviral FIV DNA using competitive PCR. Schweiz Arch Tierheilkd..

[43] Klein D, Leutenegger CM, Bahula C, Gold P, Hofmann-Lehmann R, Salmons B, Lutz H, Gunzburg WH (2001). Influence of preassay and sequence variations on viral load determination by a multiplex real-time reverse transcriptase-polymerase chain reaction for feline immunodeficiency virus. J Acquir Immune Defic Syndr.

[44] Tandon R, Cattori V, Willi B, Lutz H, Hofmann-Lehmann R (2008). Association between endogenous feline leukemia virus loads and exogenous feline leukemia virus infection in domestic cats. Virus Res.

[45] Tamura K, Stecher G, Peterson D, Filipski A, Kumar S (2013). MEGA6: molecular evolutionary genetics analysis version 6.0. Mol Biol Evol.

[46] Thompson JD, Higgins DG, Gibson TJ (1994). CLUSTAL W: improving the sensitivity of progressive multiple sequence alignment through sequence weighting, position-specific gap penalties and weight matrix choice. Nucleic Acids Res.

[47] Saitou N, Nei M (1987). The neighbor-joining method: a new method for reconstructing phylogenetic trees. Mol Biol Evol.

[48] Nei M, Kumar S, Nei M, Kumar S (2000). Phylogenetic inference: maximum parsimony methods. Molecular evolution and Phylogenetics.

[49] Felsenstein J (1985). Confidence limits on phylogenies: an approach using the bootstrap. Evolution.

[50] Hofmann-Lehmann R, Huder JB, Gruber S, Boretti F, Sigrist B, Lutz H (2001). Feline leukaemia provirus load during the course of experimental infection and in naturally infected cats. J Gen Virol.

[51] Helfer-Hungerbuehler AK, Widmer S, Kessler Y, Riond B, Boretti FS, Grest P, Lutz H, Hofmann-Lehmann R (2015). Long-term follow up of feline leukemia virus infection and characterization of viral RNA loads using molecular methods in tissues of cats with different infection outcomes. Virus Res.

[52] Johnson WE, Eizirik E, Pecon-Slattery J, Murphy WJ, Antunes A, Teeling E, O’Brien SJ (2006). The late Miocene radiation of modern Felidae: a genetic assessment. Science.

[53] Hartmann K (2011). Clinical aspects of feline immunodeficiency and feline leukemia virus infection. Vet Immunol Immunopathol.

[54] Marker L, Munson L, Basson PA, Quackenbush S (2003). Multicentric T-cell lymphoma associated with feline leukemia virus infection in a captive Namibian cheetah *(Acinonyx jubatus)*. J Wildl Dis.

[55] Harrison TM, McKnight CA, Sikarskie JG, Kitchell BE, Garner MM, Raymond JT, Fitzgerald SD, Valli VE, Agnew D, Kiupel M (2010). Malignant lymphoma in African lions *(Panthera leo)*. Vet Pathol.

[56] Kaye S, Wang W, Miller C, McLuckie A, Beatty JA, Grant CK, VandeWoude S, Bielefeldt-Ohmann H (2016). Role of feline immunodeficiency virus in lymphomagenesis - going alone or colluding?. ILAR J.

[57] Helfer-Hungerbuehler AK, Cattori V, Bachler B, Hartnack S, Riond B, Ossent P, Lutz H, Hofmann-Lehmann R (2011). Quantification and molecular characterization of the feline leukemia virus A receptor. Infect Genet Evol.

[58] Roca AL, Pecon-Slattery J, O’Brien SJ (2004). Genomically intact endogenous feline leukemia viruses of recent origin. J Virol.

[59] Coelho FM, Bomfim MR, de Andrade CF, Ribeiro NA, Luppi MM, Costa EA, Oliveira ME, da Fonseca FG, Resende M (2008). Naturally occurring feline leukemia virus subgroup A and B infections in urban domestic cats. J Gen Virol.

[60] Hoover EA, Mullins JI, Quackenbush SL, Gasper PW (1987). Experimental transmission and pathogenesis of immunodeficiency syndrome in cats. Blood.

[61] Donahue PR, Hoover EA, Beltz GA, Riedel N, Hirsch VM, Overbaugh J, Mullins JI (1988). Strong sequence conservation among horizontally transmissible, minimally pathogenic feline leukemia viruses. J Virol.

[62] Simon F, Cassaro K, Quillen P (1997). Small felid breeding project at Sao Paulo zoo. Int Zoo Yearb.

[63] Chandhasin C, Coan PN, Levy LS (2005). Subtle mutational changes in the SU protein of a natural feline leukemia virus subgroup A isolate alter disease spectrum. J Virol.

[64] Lehmann R, Franchini M, Aubert A, Wolfensberger C, Cronier J, Lutz H (1991). Vaccination of cats experimentally infected with feline immunodeficiency virus, using a recombinant feline leukemia virus vaccine. J Am Vet Med Assoc.

[65] Hofmann-Lehmann R, Holznagel E, Aubert A, Ossent P, Reinacher M, Lutz H (1995). Recombinant FeLV vaccine: long-term protection and effect on course and outcome of FIV infection. Vet Immunol Immunopathol.

[66] Lutz H, Pedersen NC, Durbin R, Theilen GH (1983). Monoclonal antibodies to three epitopic regions of feline leukemia virus p27 and their use in enzyme-linked immunosorbent assay of p27. J Immunol Methods.

